# Ideal Cardiovascular Health Metrics on the New Occurrence of Peripheral Artery Disease: A Prospective Cohort Study in Northern China

**DOI:** 10.1038/s41598-020-66578-6

**Published:** 2020-06-15

**Authors:** Dandan Wang, Qian Zhang, Anxin Wang, Shouling Wu, Xingquan Zhao

**Affiliations:** 1 Department of Neurology, Beijing Tiantan Hospital, Capital Medical University, Beijing, China; 2 China National Clinical Research Center for Neurological Diseases, Beijing, China; 3 Center of Stroke, Beijing Institute for Brain Disorders, Beijing, China; 4 Beijing Key Laboratory of Translational Medicine for Cerebrovascular Disease, Beijing, China; 5 Department of Cardiology, Kailuan Hospital, Tangshan, China

**Keywords:** Peripheral vascular disease, Lifestyle modification

## Abstract

Peripheral artery disease (PAD) is a common atherosclerotic disease which could lead to severe cardiovascular and cerebrovascular events. Previous studies have indicated the ideal cardiovascular health (ICVH) was associated with many atherosclerotic diseases and cardiovascular events. This study aimed to find out the relationship between ICVH metrics and the new occurrence of PAD. We collected information of baseline from 2010 on the seven ICVH metrics (including smoking, body mass index, dietary intake, physical activity, blood pressure, total cholesterol and fasting blood glucose); and assessed PAD by ankle brachial index among the enrolled participants. The relationship between the ICVH metrics and new occurrence of PAD was analyzed using the multivariate logistic regression in 2018. There were 214 participants were diagnosed with the new occurrence of PAD during the follow-up visit. Participants with PAD tend to be older, with a lower level of education and a higher morbidity of hypertension. Among the seven ICVH metrics, BMI seems to be the most sensitive metric to the occurrence of PAD after adjusting the other risk factors (HR (95% CI) = 0.704 (0.529–0.937), P = 0.0163). We further found out as the number of ICVH metric increased, the morbidity of PAD decreased gradually (HR (95% CI) = 0.888 (0.801–0.984), P for trend= 0.0240). There is no difference between different age and gender groups. The ICVH metrics affect the new occurrence of PAD in a Chinese population. It enhances the importance of ideal health behaviors and factors in the prevention of PAD.

## Introduction

Peripheral artery disease (PAD) is one of the most common atherosclerotic diseases around the world. It usually refers the lower limb artery atherosclerosis. In recent decades, the morbidity of PAD increases to approximately 200 million patients, especially in the older population. The prevalence increases with age from around 10% at age 65 years to 20% at age > =75 years^1^. In China, the prevalence in five surveys of populations with average ages 60–70 years varied between 2.5% and 6.9% in men and 1.7% and 10.4% in women^2^. Patients with PAD have an increased risk of cardiovascular diseases and poor prognosis^3–5^.

In 2010, the American Heart Association defined a new concept called ideal cardiovascular health (ICVH) in their *Strategic Impact Goal Through 2020 and Beyond*. This new concept is defined by the presence of both ideal health behaviors (nonsmoking, body mass index <25 kg/m^2^, physical activity at goal levels, and pursuit of a diet consistent with current guideline recommendations) and ideal health factors (untreated total cholesterol <200 mg/dL, untreated blood pressure <120/80 mmHg, and fasting blood glucose <100 mg/dL)^6^. The seven ICVH were called life’s simple seven (LSS) in these years to enhance the importance during people’ s daily life^7^, because the previous researches have reported that the ICVH was closely related with many atherosclerotic diseases, such as myocardial infarction, stroke and other vascular events^8–10^. But as we know, there is little research focus on the relationship between ICVH and the new occurrence of PAD, especially in China. So in our study, we aimed to find out the predictive value of ICVH to the occurrence of PAD, and analysis the specific characteristics among the seven ICVH metrics in the atherosclerotic disease in Chinese population.

## Results

During the 2- year follow-up, we identified 214 (5.46%) participants who had come up to the new diagnosis of PAD.

Comparing with the non-PAD participants, participants with PAD tend to be older, with a lower level of education and a higher morbidity of hypertension. Other basic characteristics such as gender, household income, medical history of diabetes mellitus and dyslipidemia and family history of stroke had no significant difference between two groups (Table 1).

**Table 1:** Basic Characteristics of Participants Regarding the Prevalence of PAD.

	PAD n = 214	No PAD n = 3702	P value
Mean age ± SD (y)	57.1 ± 14.5	53.5 ± 10.7	0.0314
Women (%,n)	82 (38.32%)	1575 (42.54%)	0.2274
Education (%,n)			0.0362
Illiteracy/Primary	29 (13.55%)	360 (9.72%)	
Middle school	103 (48.13%)	1626 (43.92%)	
High school or above	82 (38.32%)	1716 (46.35%)	
Income (%,n)*			0.2413
<¥1,000	60 (28.04%)	888 (23.99%)	
¥1,000–3,000	129 (60.28%)	2440 (65.91%)	
≥¥3,000	25 (11.68%)	374 (10.10%)	
**Previous history of disease**			
Hypertension (%,n)	65 (30.37%)	899 (24.28%)	0.0499
Diabetes mellitus (%,n)	15 (7.01%)	263 (7.10%)	1.0000
Dyslipidemia (%,n)	29 (13.55%)	421 (11.37%)	0.3216
Family history of stroke (%,n)	6 (2.80%)	113 (3.05)	1.0000

**Figure 1:**
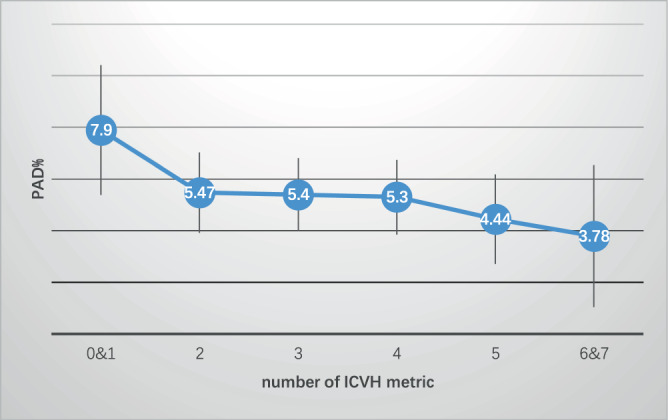
The Morbidity of PAD in different number of ICVH metric groups.

In order to find the relationship between these ICVH metrics and PAD, we in the first step took the seven ICVH metrics separately in the comparison of the occurrence of PAD. We found out that ideal BMI has the most obvious relationship with the occurrence of PAD among the seven ICVH metrics (Hazard Ration (HR) (95% Confidence Interval (CI)) = 0.684 (0.519–0.902), P = 0.0072), and the difference still exists after adjusting the relevant factors (HR (95% CI) = 0.704 (0.529–0.937), P = 0.0163) (Table 2). In the second step, we tried to find out the relationship between the ICVH score and the new occurrence of PAD, and the result showed that as the number of ICVH metric increased, the morbidity of PAD decreased gradually (HR (95% CI) = 0.888 (0.801–0.984), P for trend= 0.0240). After adjusting the age, gender and other basic characteristics, the tendency still exists significantly (HR (95% CI) = 0.882 (0.789–0.985), P for trend= 0.0264). In the third step, we then divided the participants into different subgroups by age and gender, and tried to analysis the specific value of ICVH in different age and gender subgroups. But there is no significantly difference between subgroups (Table 3). In the fourth step, in order to show the protective value of ICVH metrics to the occurrence of PAD more directly, we drew a tendency chart to show as the number of ICVH increases, the morbidity of PAD decrease gradually (Fig. 1).

**Table 2:** HRs with 95% CI of Ideal to Non-Ideal Group of Each Cardiovascular Health Metric for PAD.

Metrics	Total	
	HR(95% CI)	P value
**Smoking**		
Crude	0.996 (0.748–1.326)	0.9788
Adjusted	1.225 (0.841–1.784)	0.2912
Adjusted*	1.017 (0.710–1.456)	0.9277
**BMI (kg/m2)**		
Crude	0.684 (0.519–0.902)	0.0072
Adjusted	0.730 (0.529–1.006)	0.0547
Adjusted*	0.704 (0.529–0.937)	0.0163
**Physical activity**		
Crude	0937 (0.700–1.256)	0.6640
Adjusted	0.859 (0.616–1.196)	0.3682
Adjusted*	0.755 (0.554–1.029)	0.0751
**Diet**		
Crude	1.215 (0.879–1.681)	0.2389
Adjusted	1.547 (1.080–2.214)	0.0172
Adjusted*	1.308 (0.939–1.822)	0.1124
**Total cholesterol**		
Crude	0.924 (0.699–1.220)	0.5767
Adjusted	1.177 (0.849–1.632)	0.3281
Adjusted*	0.999 (0.749–1.332)	0.9938
**Blood pressure**		
Crude	0.507 (0.329–0.782)	0.0021
Adjusted	0.686 (0.424–1.110)	0.1250
Adjusted*	0.668 (0.423–1.055)	0.0832
**Fasting blood glucose**		
Crude	0.811 (0.607–1.083)	0.1536
Adjusted	1.014 (0.725–1.419)	0.9345
Adjusted*	0.915 (0.679–1.233)	0.5582

CI: confidence interval; BMI: body mass index.

Adjusted: The following potential confounders were adjusted for each OR: sex, age (year), education, average monthly income of the family members and family history of stroke.

Adjusted *The following potential confounders were adjusted for each OR: sex, age (year), education, average monthly income of the family members, family history of stroke, and the other six cardiovascular health metrics.

**Table 3:** HRs for PAD by the Number of Ideal Cardiovascular Health Metrics.

	Number of ideal cardiovascular health metrics							P trend
	0&1	2	3	4	5		6&7	
Cases (%)	35(7.90)	45(5.47)	54(5.40)	49(5.30)	24(4.44)		7(3.78)	
Crude OR (95% CI)	1.000	0.674 (0.427–1.006)	0.665 (0.428–1.034)	0.652 (0.416–1.022)	0.542 (0.317–0.926)		0.458 (0.200–1.052)	<0.0001
continuous	0.888 (0.801–0.984)							0.0240
Model 1 (95% CI) †	1.000	0.641 (0.404–1.018)	0.630 (0.401–0.988)	0.603 (0.378–0.964)		0.523 (0.299–0.913)	0.458 (0.196–1.072)	0.1911
continuous	0.882 (0.789–0.985)							0.0264
AGE	interaction							0.1009
**<60(year)**								
Cases (%)	24(6.78)	25(3.94)	39(5.12)	30(4.39)		10(2.42)	6(4.03)	
OR(95% CI)^‡^	1.000	0.565 (0.316–1.007)	0.742 (0.434–1.271)	0.635 (0.354–1.142)		0.351 (0.159–0.778)	0.606 (0.231–1.589)	0.1492
continuous	0.877 (0.758–1.014)							0.0761
**≧60(year)**								
Cases (%)	11(12.36)	20(10.64)	15(6.28)	19(7.88)		14(11.11)	1(2.78)	
OR(95% CI)^‡^	1.000	0.832 (0.378–1.830)	0.465 (0.203–1.066)	0.580 (0.261–1.287)		0.880 (0.375–2.064)	0.182 (0.022–1.477)	0.2228
continuous	0.895 (0.748–1.072)							0.2282
GENDER	interaction							0.8080
**Men**								
Cases (%)	27(7.18)	34(5.53)	35(5.80)	23(5.35)		11(5.82)	2(4.35)	
OR(95% CI)^§^	1.000	0.721 (0.426–1.221)	0.734 (0.434–1.241)	0.599 (0.331–1.084)		0.596 (0.282–1.261)	0.382 (0.085–1.706)	0.5238
continuous	0.873 (0.755–1.011)							0.0688
**Women**								
Cases (%)	8(11.94)	11(5.29)	19(4.79)	26(5.25)		13(3.70)	5(3.60)	
OR(95% CI)^§^	1.000	0.401 (0.153–1.051)	0.375 (0.156–0.901)	0.433 (0.186–1.010)		0.317 (0.124–0.811)	0.316 (0.097–1.024)	0.2392
continuous	0.863 (0.720–1.035)							0.1124

^†^Model 1: Adjusted for sex, age (year), education, average monthly income of every family member, and family history of stroke.

^‡^Adjusted for sex, education, average monthly income of every family member, and family history of stroke.

^§^Adjusted for age (year), education, average monthly income of every family member, and family history of stroke.

## Discussion

In our study, we found out that the ICVH metric is strongly associated with the new occurrence of PAD, that the participants who had more ICVH metrics had a fewer possibility to get the disease of PAD. To the best of our knowledge, this is the first prospective cohort study to demonstrate the predictive value of ICVH metrics to PAD in Northern China.

PAD is a common atherosclerotic disease in the elderly population, which can lead to a poor prognosis, even amputation or premature death. In the previous studies, researchers found out that when the risk factors affected the target organs and caused the atherosclerotic change, the blood supply is no longer sufficient in the target organs, and the ischemia happened gradually. To the lower extremities of body, it resulted to the disease of PAD^11^. In its initial period, patients may only suffer from intermittent claudication and pain when moving. As the ischemia and atherosclerosis aggravates, blood vessels of the whole body may be involved, and the arteries become stenosis, even occlusion, and lead to the poor outcome of cardiovascular or cerebrovascular diseases.

Previous studies have demonstrated that the ICVH metrics are strongly associated with the cardiovascular and cerebrovascular diseases. Zhang and her colleges have reported that in the same population, ICVH is related with the disease of stroke, the intracranial artery stenosis and retinal vessel calibers^12–14^. Hao and Guo found that ICVH is associated with the disease of ECAS and carotid plaque in the same population separately^15,16^. So in our study, since PAD is another vascular disease related to the atherosclerotic change, we further demonstrated the relationship and predictive value between ICVH and PAD, and got the positive answer. In other studies, Sutton reported that after controlling the PAD risk factors, including control of blood pressure, cholesterol, A1c levels, and smoking cessation, it will not only improve the morbidity and mortality, but also improve the quality of life in PAD patients^17^. Shukla studied 200 Indian diabetes mellitus patients and found out 72 of them had the evidence of PAD, and there was a significant association between PAD and duration of diabetes, waist circumference, hypertension and microvascular complications^18^. In African Americans, Collins and his colleges also got the conclusion that poor LSS is associated with the prevalence of PAD^19^, the same conclusion was reported by Gary’s team too^7^. In Parvar’s study, they reported that after a regular noninvasive management of PAD, such as achieving smoking abstinence, supervised exercise therapy and medicine therapy, the relevant major adverse cardiovascular events decreased. So medical and lifestyle management are needed in PAD patients^3^. These results are all in accordance with our study. So we can conclude that ICVH is associated with PAD and other atherosclerotic diseases. Some research has also reported that ideal LSS metrics may improve subclinical and clinical brain health outcome^20^. So, a good control and supervision of these ICVH metrics are very important to the patients healthy and prognosis. In recent years, the prevalence of ideal status was low for some metrics, such as dietary pattern, and the overall CVH status was still unsatisfactory^8^. The American Heart Association’s 2020 Strategic Impact Goal is “By 2020, to improve the cardiovascular health of all Americans by 20% while reducing deaths from cardiovascular diseases and stroke by 20%”^21^. So we should continue to measure the ICVH status and carry out lifestyle interventions to improve the ICVH status in the whole population in the next decades.

Potential limitations of our study should be discussed. First, the participants are all come from Kailuan Company living and working in Heibei province of China. So some bias may exist because of the population. We will try to continue exploring the relationship between ICVH and PAD with multi-center study in the future. Second, we excluded participants with an ABI ≥ 1.4 in our study, and it may leave some PAD participants out in the statistics. In the next study design, we will try to use the duplex ultrasound and even angiography, together with the ABI to diagnose the PAD. Third, we only used the first two-year follow-up information of the new occurrence of PAD in our study, and find out that the ICVH metric is the independent risk factor and predictive factor of PAD. Long time results of the prevalence of PAD had not collected yet. Now this study is still on its follow-up period, so we will continue focus on the study group, and find the short and long predictive value of ICVH to the PAD and other atherosclerotic diseases.

## Methods

### Study design and population

The Asymptomatic Polyvascular Abnormalities Community study (APAC) is a community-based, prospective, long-term follow-up observational study, to investigate the epidemiology of asymptomatic polyvascular abnormalities in Chinese adult. The study cohort was a sub-population of a previously described population of the Kailuan study^22^. From June 2010 to June 2011, a sample of 7000 subjects older than 40 years was randomly selected from the Kailuan cohort, and the selected method has been descripted in our previous published protocol^23^. A total of 5440 participants with no history of stroke, transient ischemic attack, or coronary disease at baseline as assessed by a validated questionnaire were included in our final APAC study. The first time follow- up visit was taken from 2012 to 2013, which is two years after the baseline visit. During the follow-up visit, we excluded 184 participants who were diagnosed PAD in the baseline and 103 participants who did not finish the follow-up visit in out study, and 1155 participants did not measure the ankle brachial index (ABI) values this time. What is more, 82 other participants were excluded in our study because of the poorly compressible leg arteries as the elevated ABI values were ≥1.40. Finally, 3916 participants were included in this study. The study was performed according to the guidelines from the Helsinki Declaration and was approved by the Ethics Committees of the Kailuan General Hospital and the Beijing Tiantan Hospital. Written informed consent was obtained from all participants.

### Assessment of cardiovascular health metrics

Information on smoking, dietary intake, and physical activity was collected via questionnaires. Body mass index (BMI) was calculated by measured weight and height during the physical examination. The measuring method of blood pressure, total cholesterol and fasting blood glucose were described in our protocol. Based on participants’ response to each relevant question and the results of these examinations, we further categorized these favorable health behaviors and factors into “ideal” and “not ideal” groups respectively according to the ICVH definition^6^. We added 1 score to every one ICVH metric, and recorded the total score of ICVH metrics from 0 to 7.

### Assessment of PAD

The ABI measurement was calculated using a standard method^24^. Systolic blood pressure was measured with a handheld 5-MHz Bidirectional Doppler probe (Hokanson MD6 Doppler with MD6VR Chart Recorder; Bellevue, Wash). Pressures in each leg were determined and the ABI was calculated separately for each leg. An ABI < 0.90 in either leg was considered as marker for the presence of a PAD, and an ABI ≥ 0.90 was considered normal. Elevated ABI values of ≥1.40 suggested poorly compressible leg arteries and were excluded from the statistical analysis^25^. We measured the ABI both at baseline and first follow-up visit to defined the disease of PAD among participants.

### Assessment of epidemiological information and vascular related risk factors

Every participant was taken a standardized questionnaire (age, gender, level of education, household income, medical history and other basic information) by our trained investigators. Hypertension was defined based on the following information alone or in combination: 1) as presence of a history of arterial hypertension; 2) using antihypertensive medication; or 3) a systolic blood pressure ≥140 mm Hg, or a diastolic blood pressure of ≥90 mm Hg. Diabetes mellitus was defined as a self-reported history, current treatment with insulin or oral hypoglycemic agents, or fasting blood glucose level ≥126 mg/dl. Dyslipidemia was defined by a self-reported history, current use of cholesterol lowering medicine, or a total cholesterol level ≥220 mg/dl or triglyceride ≥150 mg/dl or low density lipoprotein ≥160 mg/dl^22,23^.

### Follow- up and outcome assessment

We made a face- to- face interview at the participants’ first follow- up visit up to December 31, 2013, or up to the occurrence of a final event occurs (the first occurrence of cerebrovascular disease, cardiovascular disease or death). Physicians and nurses who took this follow- up are masked to the baseline data. Participant who was not able to participate in this follow- up was checked and registered according to his medical records from hospital and medical insurance^22^. During the follow-up visit, participants’ epidemiological information and vascular related risk factors were updated. Since it is an asymptomatic polyvascular abnormalities community study, the new occurrence of PAD, as well as the intracranial artery stenosis, carotid artery stenosis was all re-evaluated during the follow-ups.

### Data management and statistical analyses

The data management system is the SAS software (version 9.3; SAS Institute, Cary, North Carolina, USA). The chi-squared test was used for comparison of categorical variables and ANOVA analysis was used for continuous variables. Logistic regression was used to estimate the prevalence of PAD across health metric categories by calculating the hazard ratios and 95% confidence intervals. Other relevant risk factors were adjusted during the regression analysis. The statistics was made in 2018 and the null hypothesis was rejected for P < 0.05.
